# Multi-Modal Imaging in a Mouse Model of Orthotopic Lung Cancer

**DOI:** 10.1371/journal.pone.0161991

**Published:** 2016-09-01

**Authors:** Priya Patel, Tatsuya Kato, Hideki Ujiie, Hironobu Wada, Daiyoon Lee, Hsin-pei Hu, Kentaro Hirohashi, Jin Young Ahn, Jinzi Zheng, Kazuhiro Yasufuku

**Affiliations:** 1 Division of Thoracic Surgery, Toronto General Hospital, University Health Network, Ontario, Canada; 2 TECHNA Institute, University Health Network, Ontario, Canada; 3 Institute of Biomaterials & Biomedical Engineering, University of Toronto, Ontario, Canada; AntiCancer Inc., UNITED STATES

## Abstract

**Background:**

Investigation of CF800, a novel PEGylated nano-liposomal imaging agent containing indocyanine green (ICG) and iohexol, for real-time near infrared (NIR) fluorescence and computed tomography (CT) image-guided surgery in an orthotopic lung cancer model in nude mice.

**Methods:**

CF800 was intravenously administered into 13 mice bearing the H460 orthotopic human lung cancer. At 48 h post-injection (peak imaging agent accumulation time point), *ex vivo* NIR and CT imaging was performed. A clinical NIR imaging system (SPY®, Novadaq) was used to measure fluorescence intensity of tumor and lung. Tumor-to-background-ratios (TBR) were calculated in inflated and deflated states. The mean Hounsfield unit (HU) of lung tumor was quantified using the CT data set and a semi-automated threshold-based method. Histological evaluation using H&E, the macrophage marker F4/80 and the endothelial cell marker CD31, was performed, and compared to the liposomal fluorescence signal obtained from adjacent tissue sections

**Results:**

The fluorescence TBR measured when the lung is in the inflated state (2.0 ± 0.58) was significantly greater than in the deflated state (1.42 ± 0.380 (n = 7, p<0.003). Mean fluorescent signal in tumor was highly variable across samples, (49.0 ± 18.8 AU). CT image analysis revealed greater contrast enhancement in lung tumors (a mean increase of 110 ± 57 HU) when CF800 is administered compared to the no contrast enhanced tumors (p = 0.0002).

**Conclusion:**

Preliminary data suggests that the high fluorescence TBR and CT tumor contrast enhancement provided by CF800 may have clinical utility in localization of lung cancer during CT and NIR image-guided surgery.

## Introduction

Lung cancer is the leading cause of cancer death in the Western world, and there has been an alarming increased in incidence and deaths from lung cancer in the United States for 2014, estimated at more than 224,000 and 159,000, respectively **[1].** As per the World Health Organization, it is responsible for 1.59 million deaths worldwide annually as of 2012 **[2]**. Complete anatomical surgical resection, or lobectomy, for localized disease is the standard of care for patients with Stage I-II NSCLC yet the five-year survival rate remains 60% to 80% for stage I and only 30% to 50% for stage II non small cell lung cancer (NSCLC) according to a large series of retrospective data **[3]**. These survival rates indicate that surgeons may not be able to totally detect all primary tumor, metastasis to lymph nodes (LN) **[4]**, or ensure negative margins intra-operatively. The application of intra-operative real-time imaging has the potential to better guide surgeons to remove residual or undetected disease that may ultimately contribute to lower recurrence rates and improve overall survival.

Lung cancer is an ideal malignancy to explore intra-operative imaging because of the increasing use of low-dose CT screening, presenting surgeons with the challenge of removing small nodules neither visible nor palpable intra-operatively in greater numbers as of late **[5]**. One of the major challenges during video assisted thoracoscopic surgery (VATS) and robotic wedge resection is real-time precise localization of these lesions which can require conversion to a invasive and futile thoracotomy. Pre-operative CT-guided metal tag placement (such as a coil or hook wire) has been prevalent and successful **[6, 7]**. However, its invasive nature can result in complications including dislodgement, patient discomfort, pneumothorax or hemothorax. Therefore an improved and less invasive localization technique is necessary for the management of lung cancer. An intravenous injection of macromolecular agents that can accumulate in tumor via the enhanced permeability and retention (EPR) effect, also known as passive tumor targeting **[8],** would be an ideal preoperative localization technique method, and more importantly non-invasive.

CF800 is a novel lipid-based nano-liposomal imaging agent developed by Zheng et al. that co-encapsulates two commercially available and Food and Drug Administration (FDA) approved imaging agents, indocyanine green (ICG) for NIR fluorescence imaging and iohexol for CT imaging **[9]**. It has been engineered to allow for longitudinal and repeated dual-modal imaging using both CT and NIR imaging. CF800 can be used for contrast-enhanced pre- and intra-operative imaging following a single intravenous administration. Investigation of different animal cancer models in mice (breast, ovarian cancers) and rabbits (lung, head and neck), using CF800 have previously demonstrated effective accumulation and visualization of these solid tumors **[10–11]**. However this agent has not yet been tested on a human lung cancer animal model.

The purpose of this study was to investigate the feasibility of CF800 for disease localization and visualization using CT and real-time NIR fluorescent imaging in an orthotopic human NSCLC mouse model.

## Methods

### Cell lines

Human NSCLC cell line NCI-H460 (large cell carcinoma) was derived from primary culture, provided by Dr. Ming Tsao of the Department of Pathology, Princess Margaret Cancer Centre, Toronto. Cells were cultured and maintained as previously described by our laboratory **[12]**.

Toronto General & Western Animal Care Committee

### Animal Safety

Animal experiments and sacrifice for this study were approved by and conducted in accordance with Toronto General and Western Animal Care Committee under Animal Use Protocol 2150.13. All procedures including inoculation, imaging and injection of CF800 were conducted under anesthesia using 2–5% isofluorane inhalational gas. Criteria including muscle relaxation, toe pinch, jaw tone, respiration pattern and color of mucous membranes were used to assess the proper level of anesthesia. During recovery period, heating pads and supplemental oxygen were administered and animals were monitored every 10 minutes until able to tolerate get food and water. Animals were sacrificed in accordance with University Health Network Human Endpoint Guidelines, if human endpoints were reached such as lethargy, weight loss exceeding 20% of body weight, dyspnea, inadequate food or water intake, or inability to ambulate. Four animals died before the microCT 3-week tumor assessment from respiratory distress before euthanasia could be performed. Euthanasia was achieved using carbon dioxide gas while under anesthesia with isofluorane gas.

### Animal Model

**Fig 1:**
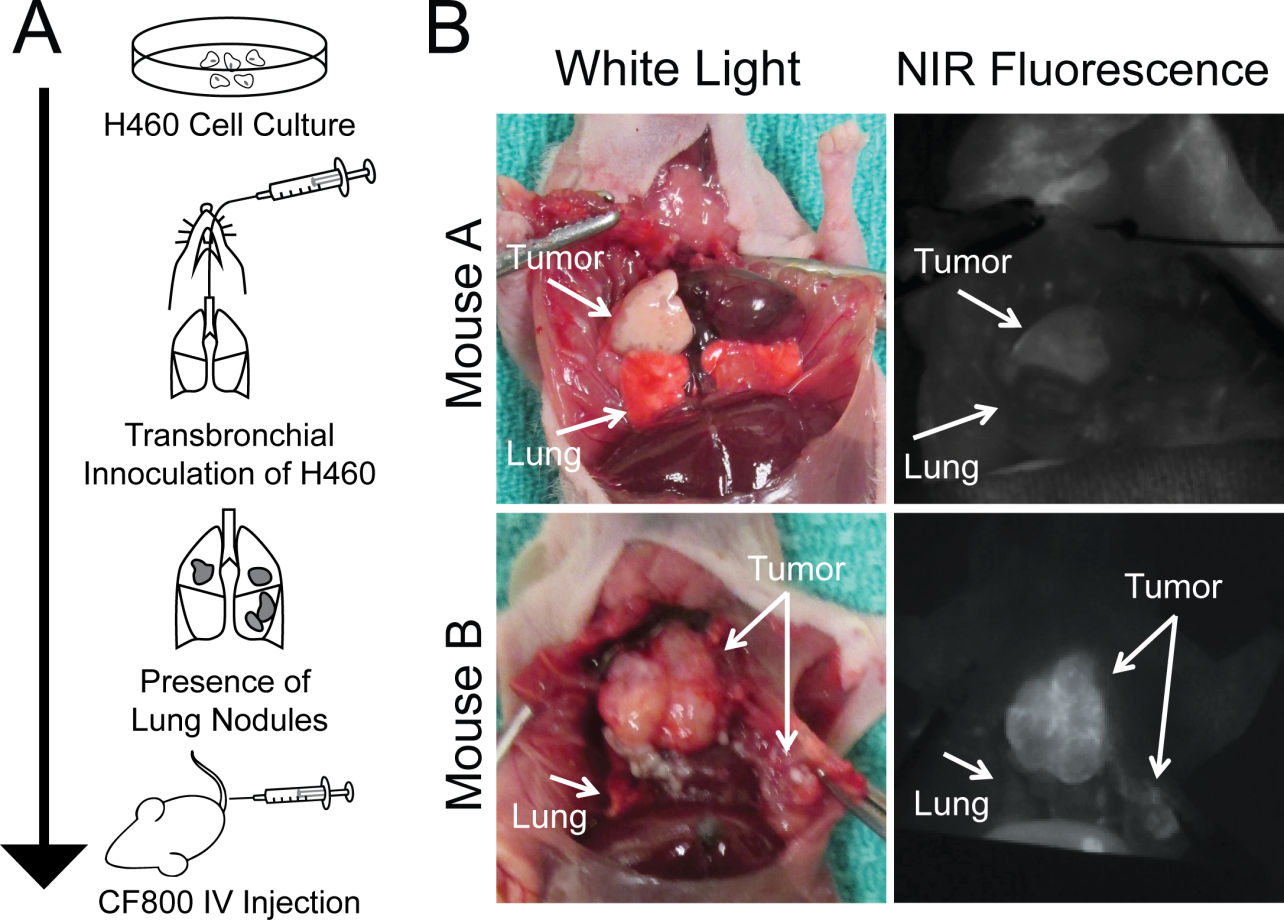
Experiment model. (A) Method of establishing orthotopic human non-small cell lung (H460) cancer house model. (B) Two representative examples of NIR ex-vivo imaging after injection with CF800.

Male athymic nude mice (Ncr-nu age 6–8 weeks; Taconic Farms Inc, Hudson, NY) were used. For the orthotopic lung cancer model, using a non-surgical, transbronchial instillation method invented by our laboratory, mice were inoculated with H460 cells as described by previous publication **[12]** (Fig 1A). Mice were monitored daily by veterinary technicians using body conditioning scoring, with particular attention to respiratory status. Tumor formation was assessed once in the mice at 3 weeks using microCT imaging with the General Electric Locus Ultra scanner (GE Healthcare, Milwaukee, WI) to ensure internal tumor growth was present. Maximum tumor size was 1.5cm.

### Imaging Agent

The CF800 liposome imaging agent was prepared and characterized as per previous publication **[9]**. Once tumor formation was detected at 3 weeks post-inoculation, each mouse (20-25g) received 200 μL of the CF800 liposome formulation injected intravenously through a tail vein catheter.

### CT and SPY Ex Vivo Imaging

After 48 hours post-CF800 injection (time of peak liposome accumulation in tumors), animals were sacrificed for ex vivo imaging. In vivo imaging was not performed in this study due to inability to successfully induce breath-hold in mice in order to obtain CT images that are free of motion-artifacts, and due to the requirement to fully expose the thoracic cavity of animals for NIR fluorescence imaging using clinical-sized open field optical imager (SPY®, Novadaq Inc., Mississauga, Ontario, Canada). Specifically, microCT imaging was completed immediately post sacrifice. An abdominal to sternum incision was performed to expose tumor bearing areas in the chest. Subsequently, a tracheostomy was created using a 20G angio catheter secured with 2–0 silk to (prevent air leaks upon lung inflation). The SPY® imaging system was used to detect fluorescent tissue within the thoracic cavity in real time (Fig 1B).

### Histo-pathological evaluation

**Fig 2:**
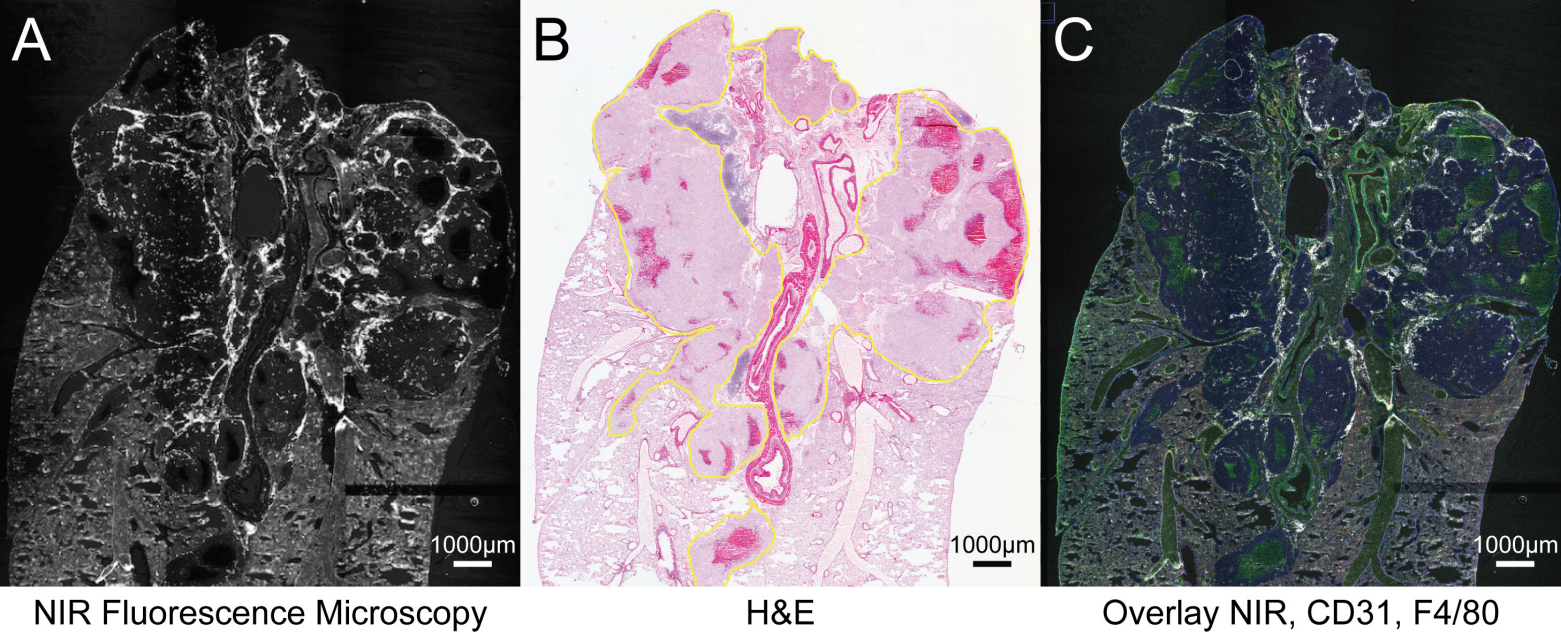
CF800 localization in tumor. (A) Histological section of tumor viewed with Tissuescope under NIR, (B) (H&E), (C) overlay of NIR, and immunocytochemical markers CD31 and F4/80. Areas of NIR signal detected with Tissuescope correlates with malignant cells on H&E staining. Malignant cells outlined in yellow.

After excision of lung and tumor (in situ), tissue samples were frozen in OCT, sectioned and stained with hematoxylin and eosin (H&E) for observation under white field microscopy. Immunocytochemical analysis with F480 and CD31 was performed to identify macrophages and vessel endothelium. Fluorescent microscopy of histological sections was observed using Tissuescope (TS4000, Huron Technologies), (Fig 2). Automated computer image analysis software for pathology, *Definiens TissueStudio* (Definiens AG, Munich Germany) was used to quantify and/or overlay NIR signal with F4/80 and CD31 markers in regions of interest (ROI) identified as tumor, necrosis and lung parenchyma as seen on corresponding H&E images.

### CT and SPY Image Analysis

Using *Microview* software, CT based image analysis was performed as per previously described methods **[9]**. The tumor volumes were contoured using a semi-automated threshold based method **[13]** to generate volume-of-interest (VOI) for subsequent analysis. The mean and standard deviation of the voxel signal distribution within each VOI was calculated.

Tissue fluorescence detected by SPY was quantified using *ImageJ* (National Institute of Health, Bethesda, Maryland). Fluorescence intensity was measured by manually placing ROIs on structures of interest (tumor) and background (lung parenchyma). Fluorescence mean intensity and standard deviation of the signal level were then extracted. Tumor-to-background ratio (TBR) was calculated using the mean signal intensity of tumor and corresponding mean signal intensity of background for each mouse.

### Statistical Analysis

Differences between TBRs were compared using two-tailed student’s T-test with 95% confidence interval. Due to the preliminary nature of this study and small sample size, mean fluorescence signals and CT tumor signals are stated as means with standard deviations.

## Results

### Tumor Localization and Visualization Using NIR Fluorescence

**Fig 3:**
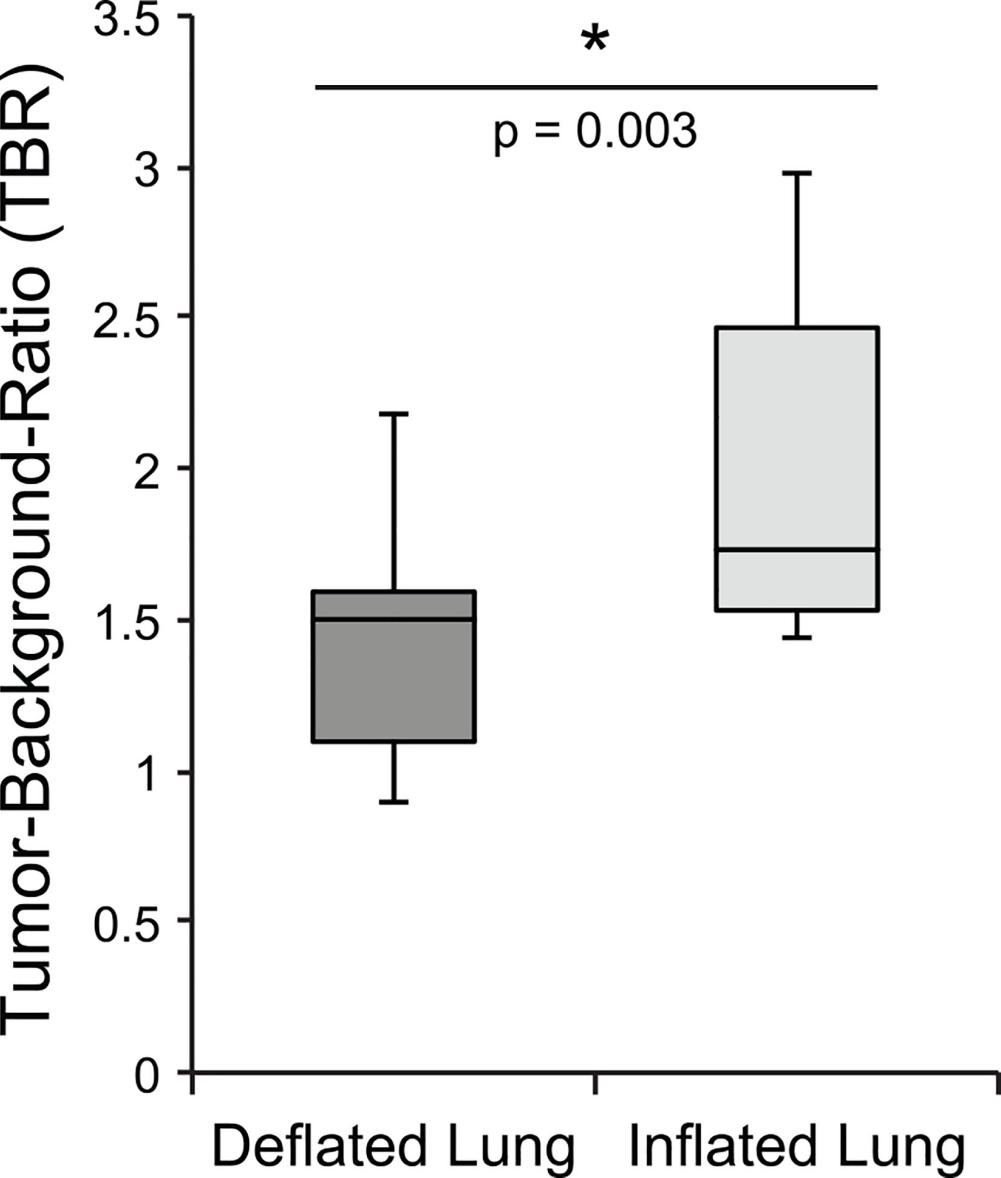
Comparison of tumor background ratio in inflated and deflated lung. Boxplot demonstrating a statistically significant increase in tumor-background ratio with inflated lung compared to deflated lung, n = 7 (p = 0.003).

Tumor fluorescence was evaluated using the open-field NIR fluorescence imaging system customized for imaging ICG (excitation: 785nm, emission: 820nm). The laser beam was aimed at the open thoracic cavity bearing tumor laden lung. A significant degree of background fluorescence was detected from collapsed lung (atelectasis) in the pilot study. For that reason, we performed post-mortem tracheostomy in order to effectively control inflation and deflation of the lung. Upon inflation, the fluorescent signal of the background lung demonstrated a significant decrease allowing for clearer delineation of normal lung from malignant tissue. Therefore an appreciably higher TBR was achieved when the lung was inflated, 2.00 ± 0.58 (min 1.41, max 2.98) compared to the deflated state 1.42 ± 0.38 (min 0.89, max 2.18), p = 0.003, n = 7 (Fig 3).

Mean fluorescent signal of tumor alone (n = 13) was 49.0 ± 18.8 arbitrary units (AU), (min 28.3, max 100.7), compared to deflated lung alone 34.6 ± 11.1 and inflated lung alone 23.4 ± 5.6, indicating a significantly greater and more variable CF800 uptake in tumor than surrounding lung.

### Histological Assessment of CF800 Localization

**Fig 4:**
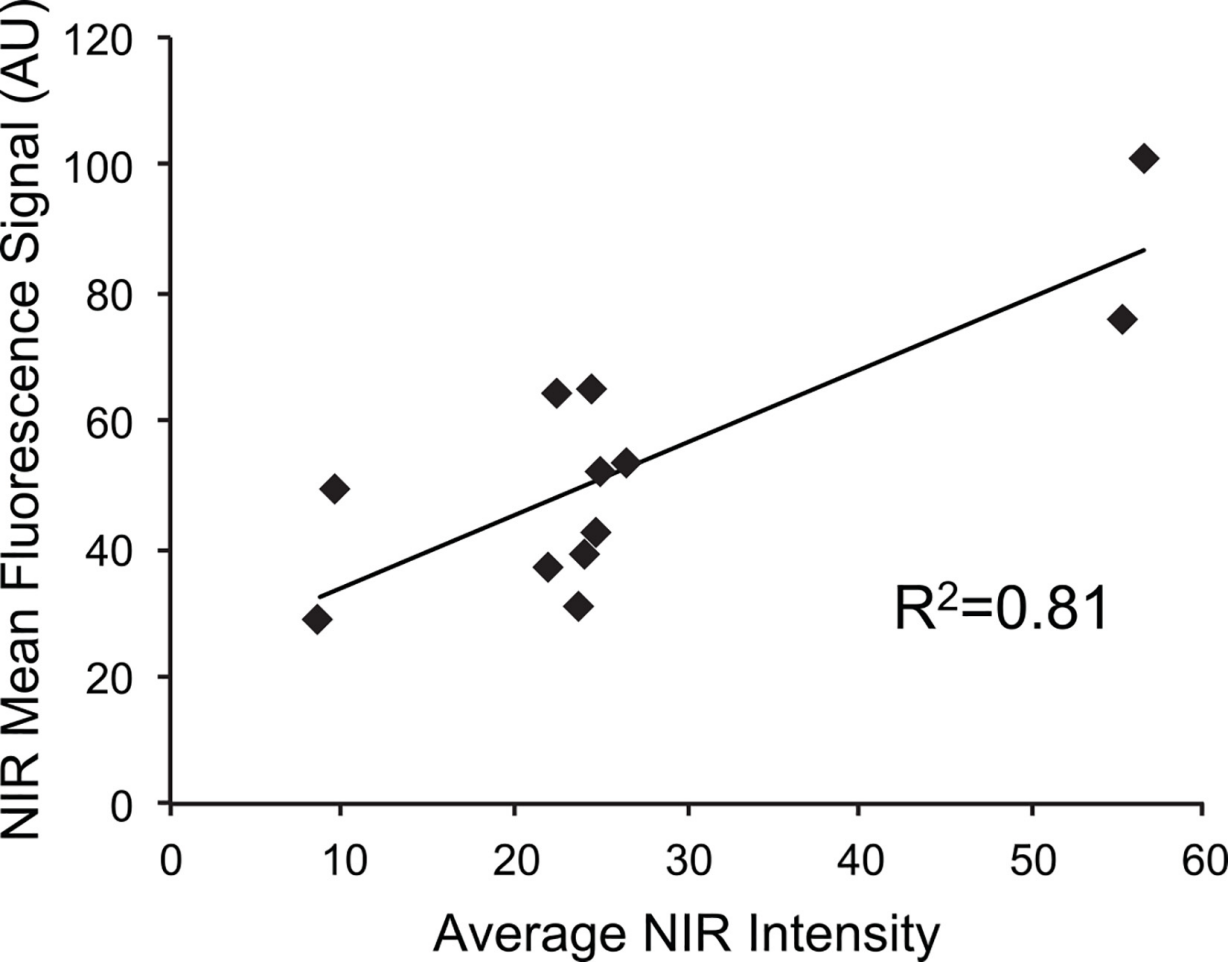
Correlation between NIR intensity and NIR mean fluorescence. Positive correlation between NIR intensity and NIR mean fluorescence. SPY can be used as a quantitative tool to accurately measure ICG liposome accumulation. Positive correlation (R^2^ = 0.81) exists between measured SPY fluorescence image signal and average NIR intensity of tumor on histological section.

he fluorescence signal detected using a high resolution fluorescence histology imager correlated with the presence of tumor as confirmed with conventional H&E staining. Qualitatively, NIR signal concentrated greatest in tumor periphery (Fig 3). Areas of tumor necrosis displayed almost no NIR signal. We further correlated the gross fluorescent signal observed using the open-field NIR fluorescence imager to that obtained from the fluorescence detected using the high resolution histology scanner on sections from the same tumor and a strong positive correlation (R^2^ = 0.81, n = 11) was found (Fig 4).

**Fig 5:**
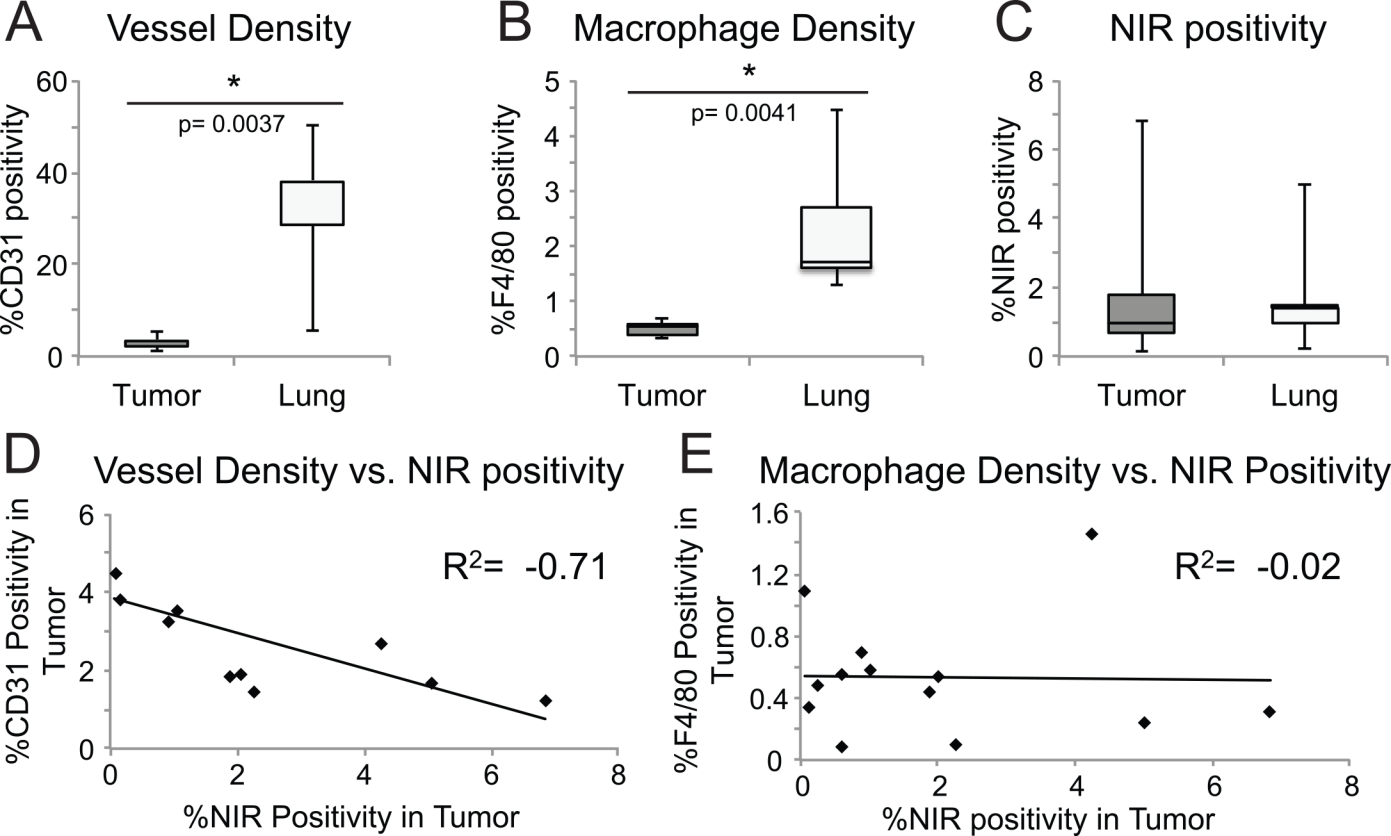
Histology based characterization. Histology of tumor and normal lung compares boxplots of (A) vessel density p = 0.0037 (A), and (B) macrophage density p = 0.0041 (B) student’s t-test demonstrating they are lower in tumor compared to lung. However (C) NIR positivity is overall slightly increased and more variable in tumor. Correlation between vessel density and NIR positivity (D) is strongly negative whereas macrophage density (E) is not correlated to NIR positivity.

Histology-based characterization of tumor and normal lung demonstrates the vessel density (CD31 staining) and macrophage density (F4/80 staining) is lower in tumor compared to the healthy lung parenchyma, yet NIR fluorescence positivity is overall higher in tumor compared to lung parenchyma (Fig 5). Specifically, tumor NIR intensity correlated negatively with tumor vessel density (R^2^ = -0.71), but not with macrophage density (R^2^ = -0.012) suggesting that neither perfusion nor macrophage uptake accounted for CF800 retention (Fig 5). Further, we compared vascular density between tumors of different size due to the observation that signal intensity was often greater in the larger/invasive tumors, hypothesizing that greater perfusion would induce improved nanoparticle delivery and accumulation, and thus greater fluorescence signal. However we found that smaller/solitary tumors had greater vessel density but lower fluorescence signal. Although this difference was not statistically significant, it suggests that tumor perfusion alone is likely to only influence nanoparticle delivery but not accumulation. In fact, in this human NSCLC model, it is apparent that vascular density and perfusion do not directly dictate the intratumoral deposition of nanoparticles, but rather the degree of disease progression and vascular leakiness are determinant factors for liposome accumulation.

### 3D Localization of Lung Cancer using CT Imaging

**Fig 6:**
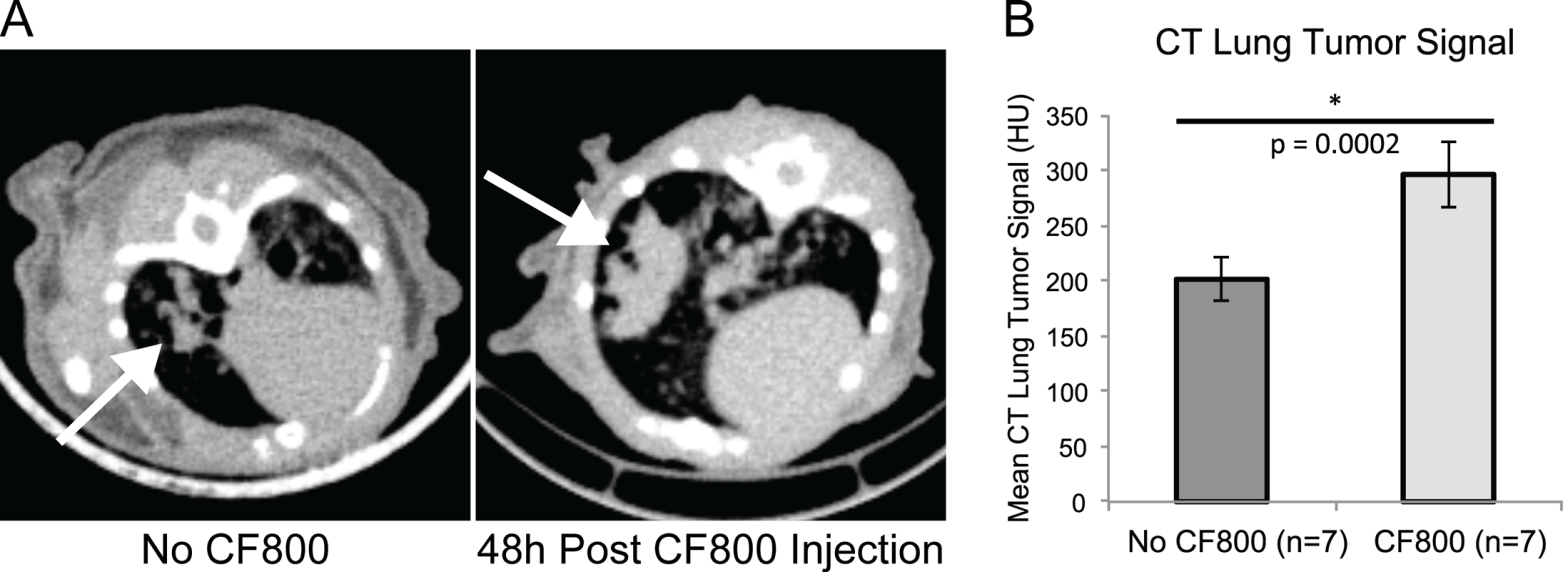
Representative CT axial images of mouse lung. (A) CT axial images of mouse lung with no CF800 injection (left) and at 48h post-CF800 injection (right). The arrows point to the location of the lung tumor. Both images are displayed at the same window and level. Note the higher CT signal observed in the tumor of the mouse injected with the CF800 agent. (B) Bulk tumor analysis demonstrating CF800 injected mice have a higher tumor signal than non-injected mice, p = 0.0002.

Ex vivo CT imaging was performed 48 hours post CF800 liposome injection immediately after animal sacrifice, before opening the chest cavity, in order to minimize anatomical deformation and also avoid motion artifacts. As expected, CF800 accumulated in lung tumor, demonstrating a statistically higher mean signal (311 ± 55 HU, n = 7) compared to control lung tumors of similar size without liposome injection (201 ± 60 HU, n = 7,) (Fig 6). This demonstrates the utility of CF800 for pre-operative or intra-operative CT or cone-beam CT based 3D localization and delineation.

## Comment

The current method for lung nodule localization during minimally invasive thoracic surgery relies on CT guided wire or coil placement and is a thorough and efficient means of excising lung nodules. However systemic administration of nano-liposomes that localize in malignant tissue could potentially permit real-time intra-operative imaging in addition to effective pre-operative CT-based surgical planning. The added benefit of using an injectable contrast agent compared to wire or coil placement is a much lower level of invasiveness thereby eliminating risks of pneumothorax and hemothorax. Tumor identification and delineation from healthy lung tissues was successfully demonstrated in these animal models using both CT and NIR fluorescence based imaging. The difficulty however lies in detecting the smaller and more clinically relevant animal lung tumor nodules that fluoresced less, compared to the less clinically relevant larger or invasive tumors that fluoresced higher. Our models also exhibited nodules already extending to the pleura–this does not mimic the real clinical dilemma of localizing non-visible/non-palpable nodules deep within the parenchyma nor does it address the limit of fluorescence depth penetration.

Tumors typically exhibit accumulation of the liposome due to the EPR effect that arises from leaky vasculature within tumors **[8]**. EPR itself is a highly variable phenomenon with large inter and intra-tumoral differences thus contributing to the heterogeneity of fluorescence signal intensity we observed with our model. Solid tumors especially when small tend to demonstrate hypervascularity **[8]**. As demonstrated in our results, small tumors exhibited higher vascular density compared to the larger tumors, however this does not dictate liposome accumulation or positively correlate to vascular permeability. Extravasation assays would be required to verify vessel permeability and to determine if increasing nodule size alone corresponds to more efficient passive tumor targeting.

Mouse lung parenchyma proved challenging to image compared to previous animal models with subcutaneous or peritoneal tumor implants. However we found that by changing the air/tissue interface through inflation (though full inflation is not a clinically realistic option in minimally invasive surgery) we could control the background signal intensity and effectively increase the TBR. This phenomenon requires a more comprehensive investigation of the fluorescence signal from lung parenchyma upon alveolar thinning and airway expansion that may ultimately help optimize lung imaging for the detection of tumor or malignant lymph nodes.

As expected, the CT signal of lung tumor increased significantly after CF800 injection. Although CT is capable of detecting lung nodules up to less than 5mm without any imaging agents **[14]**, the added contrast offered by CF800 may allow for detection of sub-millimeter or micro-metastatic disease and function as an adjunctive clinical staging modality tool to PET or PET/CT.

The use of NIR fluorescence has gained much momentum over the last few years, and many advances have been made in the design and development of new imaging probes for fluorescence-guided tumor resection. We believe that our EPR-targeted tumor imaging approach using a nano-sized liposome imaging agent provides an alternative broad tumor imaging solution to previously published small molecule based single modality fluorescence imaging agents. For example, studies have demonstrated effective visualization of primary tumors and metastatic deposits such as folate conjugated to fluorescein isothiocyanate which is targeted to folate receptor-α, which is often but not always expressed in ovarian cancer **[15]**. Other studies selectively label tumors using telomerase dependent adenovirus (OBP-401) that expresses the green fluorescent protein in cancer cells only, and maintain expression to allow for detection of cancer recurrence **[16, 17, 18]**. The data has suggested that adenoviral-GFP labeling tumors can enable far more superior for resection of malignant tissue with regards to disease free survival, compared to conventional bright light surgery when using genetic labeling methods **[19]**. Furthermore there are reports of fluorescence guided surgery in combination with OBP-401 as being curative for soft tissue sarcoma in an orthoropic mouse model **[20]**. These features are unique because they are not within the capabilities of non-genetic probes, however some argue that the disadvantage of telomerase dependent adenoviruses is that telomerase is only present in up to 85% of primary tumors **[21]**.

Once a nano imaging agent is chosen, much debate still exists between the effectiveness of passive versus active tumor targeting. Based on the experience gained from using liposomes for drug delivery, tumor cell-surface targeted active targeting strategies are limited because they require EPR-mediated extravasation prior to binding **[22]**. However, in tumors that exhibit limited EPR but high vascularity, a viable strategy to improve the passive delivery and accumulation of nanoparticles to tumors, regardless of sizes and vascular permeability, would consist in actively targeting liposomes to specific epitopes expressed on the tumor vasculature **[22]**. This strategy has previously been shown to successfully improve tumor deposition of liposomes by our group **[23]**. Its advantage consists in the fact that vascular targets are easily accessible and positively correlate to hypervascularity. l. However, over time, other groups reported that passive targeting has led to overall more efficient retention in tumors **[24]**. For intra-operative imaging, this may prove to be less useful than for drug delivery or longitudinal imaging studies but it should not discount the potential of imaging agents that rely on the EPR phenomenon, as this is a more universal tumor targeting means than any actively targeted moiety. For example, there is currently a clinical trial evaluating the potential for active tumor targeting using an injectable folate-fluorescein conjugate (EC17) to detect folate receptor alpha (FRA) positive lung tumors using an intra-operative imaging system **[25]**. However, less than 75% of lung adenocarcinoma and less than 13% of squamous cell carcinoma express FRA **[26]**. For this reason, passive targeting may prove be more inclusive when approaching thoracic malignancies.

Although coil/wire localization remains the gold standard for lung nodule localization in minimally invasive thoracic surgery, nano-liposome imaging agents could potentially improve the detection of subclinical disease, obtain negative margins, or detect metastatic LNs missed through other modalities. In conclusion we were able to demonstrate localization of CF800 in orthotopic lung cancer tumors and successfully visualize them using a NIR fluorescence imager and CT.

## Abbreviations

AU: Arbitrary unit
CT: Computed tomography
EPR: Enhanced permeability and retention
FDA: Food and Drug Administration
FRA: Folate receptor alpha
H&E: Hematoxylin &eosin
HU: Hounsfield unit
ICG: Indocyanine green
LN: Lymph nodes
NIR: Near infrared
NSCLC: Non small cell lung cancer
ROI: Region of interest
TBR: Tumor-to-background ratio
VATS: Video assisted thoracoscopic surgery
VOI: Volume-of-interest
